# Redox proteomics and physiological responses in *Cistus albidus* shrubs subjected to long-term summer drought followed by recovery

**DOI:** 10.1007/s00425-014-2221-0

**Published:** 2014-12-13

**Authors:** Ricard Brossa, Marta Pintó-Marijuan, Rita Francisco, Marta López-Carbonell, Maria Manuela Chaves, Leonor Alegre

**Affiliations:** 1 Departament de Biologia Vegetal, Facultat de Biologia, Universitat de Barcelona, Avda. Diagonal 643, 08028 Barcelona, Spain; 2 Molecular Ecophysiology Lab. (LEM), Instituto de Tecnologia Química e Biológica, Universidade Nova de Lisboa, 2780-901 Oeiras, Portugal

**Keywords:** Antioxidants, Photosynthesis, Plant hormones, Protein carbonylation, Water stress

## Abstract

**The interaction between enzymatic and non-enzymatic antioxidants, endogenous levels of ABA and ABA-GE, the rapid recuperation of photosynthetic proteins under re-watering as well the high level of antioxidant proteins in previously drought-stressed plants under re-watering conditions, will contribute to drought resistance in plants subjected to a long-term drought stress under Mediterranean field conditions.**

This work provides an overview of the mechanisms of *Cistus albidus* acclimation to long-term summer drought followed by re-watering in Mediterranean field conditions. To better understand the molecular mechanisms of drought resistance in these plants, a proteomic study using 2-DE and MALDI-TOF/TOF MS/MS was performed on leaves from these shrubs. The analysis identified 57 differentially expressed proteins in water-stressed plants when contrasted to well watered. Water-stressed plants showed an increase, both qualitatively and quantitatively, in HSPs, and downregulation of photosynthesis and carbon metabolism enzymes. Under drought conditions, there was considerable upregulation of enzymes related to redox homeostasis, DHA reductase, Glyoxalase, SOD and isoflavone reductase. However, upregulation of catalase was not observed until after re-watering was carried out. Drought treatment caused an enhancement in antioxidant defense responses that can be modulated by ABA, and its catabolites, ABA-GE, as well as JA. Furthermore, quantification of protein carbonylation was shown to be a useful marker of the relationship between water and oxidative stress, and showed that there was only moderate oxidative stress in *C. albidus* plants subjected to water stress. After re-watering plants recovered although the levels of ABA-GE and antioxidant enzymes still remain higher than in well-watered plants. We expect that our results will provide new data on summer acclimation to drought stress in Mediterranean shrubs.

**Electronic supplementary material:**

The online version of this article (doi:10.1007/s00425-014-2221-0) contains supplementary material, which is available to authorized users.

## Introduction

*Cistus albidus* (Rockrose) is a semi-deciduous shrub that is highly acclimated to summer Mediterranean drought conditions. Gas exchange regulation, cell redox homeostasis and hormonal regulation responses to drought have been previously studied in this shrubs (Munné-Bosch et al. 2001; Jubany-Marí et al. 2009; Galle et al. 2011); however, currently, large gaps still remain in the understanding of the different regulatory networks that control complex plant responses to the dynamic environment in which it grows (Luhua et al. 2013).

Plant acclimation to stress is associated with profound changes in leaf proteome, thus, proteomic studies can significantly contribute to unraveling the possible relationship between protein modifications and plant stress acclimation (Kosová et al. 2011), namely those that result from changes in the cell redox state. In recent years, several proteomic studies have been performed on various species under water deficit conditions such as poplar, oak, rice, sunflower and grapevine (Costa et al. 1998; Salekdeh et al. 2002; Hajheidari et al. 2005; Ali and Komatsu 2006; Jorge et al. 2006; Plomion et al. 2006; Vincent et al. 2007; Bonhomme et al. 2009b; Fulda et al. 2011; Sergeant et al. 2011) and it has been showed that proteomic analysis may reveal subtle differences in drought stress responses that are not evident from mRNA measurements (Hajheidari et al. 2005). However, to the best of our knowledge, this is the first report to study simultaneously the dynamics of the leaf proteome alongside photosynthesis, antioxidants and plant hormones in *C. albidus* shrubs growing in their natural habitat and subjected to long-term summer drought stress.

Proteins are the main cellular target of reactive oxygen species (ROS), accounting for 68 % of the total oxidized molecules in plant cells (Rinalducci et al. 2008). Carbonyl group formation in proteins is the major form of irreversible oxidation, leading to the inactivation or breakdown of proteins (Levine 2002) and is also known to contribute to cellular damage and biotic stress (Møller et al. 2007). Protein carbonylation can easily be monitored by means of conjugation with dinitrophenylhydrazine followed by immunodetection or spectrophotometric readings (Levine et al. 1994). In spite of the fact that these methods only focus on one type of protein modification without taking into consideration all redox protein networks, the assessment of protein carbonylation can be used as an oxidative stress marker in plant cells (Johansson et al. 2004).

At the physiological level, the regulation of photosynthesis is a key factor in plant acclimation and survival during drought periods (Chaves et al. 2002). The downregulation of photosynthesis could be due to a limitation of stomatal conductance and such results are typical in plants which are more tolerant in mild-to-moderate water deficit conditions. In addition, as the stress progresses, downregulation of the proteins involved in the photosynthetic process can be observed. Under stress conditions, the limitation of photosynthesis induces oxidative stress (Apel and Hirt 2004). An imbalance of ROS means that an effective antioxidant response is crucial to maintain redox homeostasis (Jubany-Marí et al. 2010a) and in most extreme circumstances to guarantee plant survival. Soluble ascorbate and glutathione are the main non-enzymatic antioxidants in plants responsible for ROS detoxification (Noctor and Foyer 1998; Smirnoff 2000;). ROS scavenging enzymes such as superoxide dismutase (SOD), ascorbate peroxidase (APX), catalase (CAT), glutathione peroxidase (GPX) and peroxiredoxin (PrxR) are also essential in maintaining redox homeostasis. Moreover, almost all stresses induce the production of a group of HSPs that function as chaperones thereby conferring plant resistance to stress (Al-Whaibi 2011).

The plant hormone abscisic acid (ABA) plays a crucial role in plant responses to environmental stress. Plants must constantly adjust ABA levels to respond to changing physiological and environmental conditions (Nambara and Marion-Poll 2005). The level of this hormone is regulated by the relative rates of biosynthesis, catabolism, conjugation and its distribution throughout the plant. In particular, ABA increases when plants are subjected to water stress and decreases when rescued from stress conditions, thus ABA metabolism is a crucial aspect of the plant response to conditions of stress (Xiong and Zhu 2003). Moreover, *C. albidus* plants showed an increase in abscisic acid glucose ester ABA-GE at the beginning of summer that preceded the increase of free ABA (López-Carbonell et al. 2009); this increase in ABA-GE could have provided a source of ABA after subsequent hydrolysis (Sauter et al. 2002) ABA was shown to increase the expression and the activity of ROS network genes such as CAT1, APX1, glutathione reductase (GR1) (Zhang et al. 2006) and cytosolic Cu/ZnSOD, as well as APX and GR in leaves of maize (Hu et al. 2005). Jasmonates are also key modulators of plant responses under biotic and abiotic stress. Moreover, redox state regulation is important in responses to ABA during drought. Jasmonic acid (JA) and methyl jasmonate (MeJA) levels increase under water stress in several plant species (Gapper et al. 2002; Pedranzani et al. 2007; Jubany-Marí et al. 2010b) and in addition, ABA and JA participate in the complex network of plant resistance to drought-oxidative stress (Foyer and Noctor 2009). Crosstalk between ABA and JA which regulates ascorbate and glutathione levels (Sasaki-Sekimoto et al. 2005; Wolucka et al. 2005; Piotrowska et al. 2010) has been demonstrated. However, there is a lack of knowledge about the relationship between these hormones and the dynamics of the redox proteome in *C. albidus* shrubs subjected to a long-term period of drought followed by recovery.

As proteins are the main cellular targets of oxidative stress, proteomic studies concomitant with physiological responses can significantly contribute to unraveling the possible relationship between water-oxidative stress and protein modifications in acclimation responses of plants to drought conditions.

The aim of this work was to study the leaf proteome, physiological processes, redox homeostasis and plant hormones in the network of the acclimation responses of *C. albidus* plants to long-term summer drought followed by recovery. In addition, the possible role of the plant hormones, ABA, ABA-GE, (PA) and DPA, and JA in hormones-antioxidants network is studied.

## Materials and methods

### Plant material and field growth conditions

The experiments were conducted on the Mediterranean semi-deciduous shrub, *C. albidus*, grown under Mediterranean conditions in the Experimental Fields of the Faculty of Biology at the University of Barcelona. Experiments were carried out with 50 homogenous plants, 1 year old, from June 21 to December 1. Plants were divided into two treatments: well-watered (WW) plants, which comprised of 25 plants watered to field capacity throughout the experiment; and water-stressed (WS) plants made up of another 25 plants that did not receive water until the recovery period (from November 5 to December 1). To prevent water contributions from rainfall, the WS plot was equipped with a mechanical PVC rain shelter (Grup Sabater, Argentona, Spain) fitted with a rain sensor that allowed for the immediate and automatic closure of the rain shelter sheets when the first rain drops were detected, but which remained open when rain was not detected. During the recovery period, WS plants were watered to field capacity to study the recovery of plants after water deficit.

**Fig. 1:**
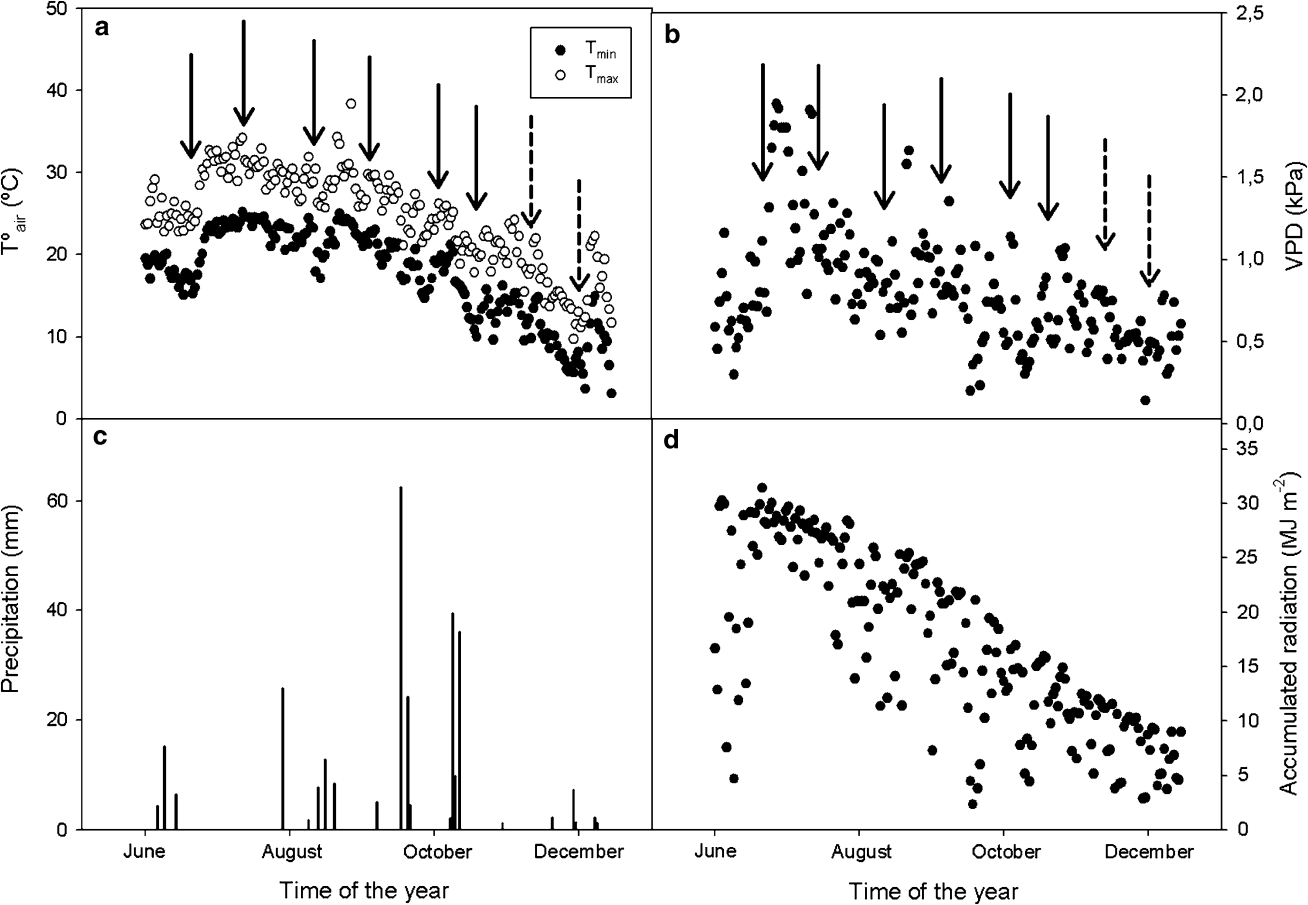
Environmental conditions during the measurement period. **a** Air temperature (*T*º_air_,  °C) maximum diurnal temperature (*closed circle*) and minimum diurnal temperature (*open circle*). **b** Maximum diurnal vapor pressure deficit (VPD). **c** Precipitation (*vertical bars*). **d** Accumulated radiation (MJ m^−2^). *Arrows* indicate data corresponding to sampling days. *Dotted arrows* indicate data corresponding to sampling in the recovery period

The environmental conditions (Fig. 1) were monitored throughout the study with a weather station (KIPP and ZONEN, Delft, The Netherlands). During the experiment, plants were exposed to maximum air temperatures 32 °C (Fig. 1a), a maximum diurnal vapor deficit (VPD) which was recorded in July (ca. 2 kPa) (Fig. 1b), precipitation was scarce (Fig. 1c), and the accumulated radiation was ca. 30 MJ m^−2^ (Fig. 1d). This period of drought was followed by a decrease in air temperatures (T_air_—maximum of ca. 20 °C in November with progressively lower temperatures thereafter), a VPD average of 0.5 kPa, a progressive decrease in accumulated radiation and an increase in precipitation, with rainy days registered in October and November. Arrows in Fig. 1 indicate data corresponding to sampling days throughout the experiment. Dotted arrows indicate data corresponding to sampling for water recovery.

Plant water status, leaf gas exchange and fluorescence, protein identification, protein carbonylation, plant hormones, antioxidants of low molecular weight such as ascorbate (AsA), dehydroascorbate (DHA), glutathione (GSH) and oxidized glutathione (GSSG) were analyzed in leaves taken from plants situated 5–15 cm from the apex at midday and at maximum diurnal incident photon flux density (PFD) on clear sunny days every 3–4 weeks. For biochemical studies, samples were immediately frozen in liquid nitrogen and stored at −80 °C until analysis was carried out.

### Plant water status and leaf mass area

Leaf relative water content (RWC) was calculated as follows: RWC (%) = (FW − DW)/(TW − DW), where FW is the fresh weight, TW is the turgid weight after re-hydrating the samples for 24 h at 4 °C in darkness, and DW is the dry weight after oven-drying samples at 80 °C until constant weight.

The leaf area (LA) was measured with a flatbed scanner (Model CX-5400 Epson Stylus) and processed using the leaf area measurement program (Version 1.3, The University of Sheffield, 2003) image analyzer software. The leaf mass area (LMA) was calculated as LMA = DW/LA. Values given are the means of three independent samples per shrub (with four leaves per sample).

### Leaf gas exchange and fluorescence measurements

Leaf gas exchange measurements were performed using an open IRGA LI-Cor 6400 system (LI-Cor Inc., Lincoln, NE, USA) equipped with a leaf fluorescence chamber. Measurements were always performed on sun-exposed leaves located at least 10 cm from the branch apex at saturation photosynthetic photon flux density (PPFD, 1,200 μmol m^−2 ^s^−1^), ambient air temperature, and 370 μmol mol^−1^ of CO_2_. The analyzed parameters were as follows: assimilation rate (A), transpiration rate (E), stomatal conductance (g_s_), leaf-to-air vapor pressure deficit (VpdL), and leaf temperature (*Tº*_leaf_).

Chlorophyll fluorescence measurements were made on the attached leaves which were dark adapted for 25 min using LI-Cor dark-adapting leaf clips. Maximum quantum efficiency of PSII photochemistry (*F*_v_/*F*_m_) was monitored.

### Protein extraction

Leaves from five sampling days (June 21, August 9, September 3, October 20 and November 11) were selected for protein extraction following the procedure described by (Xu et al. 2008). Briefly, leaf samples (150 mg FW) were ground in liquid nitrogen with a mortar and pestle; afterwards, they were incubated with 2 ml of precipitation solution [10 % trichloroacetic acid (TCA) and 0,1 % dithiothreitol (DTT) in acetone] for 2 h at −20 °C. The precipitated proteins were pelleted and washed with ice-cold acetone containing 60 mM DTT. The pellet was vacuum-dried and re-suspended in 7 M urea, 2 M thiourea, 0.4 % (v/v) Triton-X100, 4 % (w/v) CHAPS, 1 % (v/v) IPG buffer and 60 mM DTT. The insoluble tissue was removed by centrifugation at 15 000*g* for 10 min. Protein concentration was determined using the 2DE Quant Kit (GE Healthcare).

### Two-dimensional PAGE

An IPGPhor apparatus (GE Healthcare, Piscataway, NJ, USA) was used for isoelectric focusing (IEF) with immobilized 13 cm (IPG) strips with a non-linear pH gradient (pH 3.0–10). The IPG strips were loaded with a 50 µg sample protein in a total volume of 250 μl of rehydration buffer [7 M urea, 2 M thiourea, 0,4 % (v/v) Triton-X100, 4 % (w/v) CHAPS, 1 % (v/v) IPG buffer, 60 mM DTT]. The voltage settings for the IEF were 30 V, 12 h (re-hydration step); 100 V, 150 Vh (S2); 250 V, 250 Vh (S3); 1,000 V, 1,500 Vh (S4); 2,500 V, 2,500 Vh (S5); 8,000 V, 30 min (Gradient, S6); 8,000 V, 32,000 Vh (S7); 100 V (S8).

Following IEF, the gel strips were denatured twice (50 mM Tris–HCl pH 8.8, 6 M urea, 30 % glycerol, 2 % SDS) over a period of 15 min, first with 1 % DTT followed by a second incubation with the same buffer containing 2.5 % iodoacetamide.

The second dimension electrophoresis was performed on a 12.5 % gel using an electrophoresis unit (Bio-Rad Laboratories Inc.). Gels were stained with Flamingo reagent (Bio-Rad Laboratories Inc.). Briefly, each gel was fixed with 200 ml of 40 % Ethanol, 10 % acetic acid solution and stained with 100 ml of 1/10 diluted Flamingo reagent. Stained gels were scanned on a Fujifilm FLA-5100 (Fuji Storm) using a 532 nm laser at 600 V.

### Two-dimensional image processing and statistical analyses

Gel images were analyzed with Progenesis (version 4.01; Non-linear) software. Image analysis included the following procedures: spot detection, spot measurement, background subtraction, and spot matching. Only spots that were detected on all three replicate gels were analyzed further. To correct the variability due to staining, the spot volumes were normalized as a percentage of the total volume of all spots on the gel. Data were subjected to analysis of variance (ANOVA; *P* < 0.05) to monitor sampling dates and water-stress effects.

### Protein identification

Protein identification was performed at the Proteomics Platform of Barcelona Science Park, University of Barcelona. The gel spots were excised and washed with 30 % acetonitrile (ACN) in 50 mM ammonium bicarbonate prior to DTT reduction and iodoacetamide alkylation. Trypsin (Trypsin Gold, Promega) was used, 80 ng/sample, for digestion at 37 °C overnight. The resulting peptides were extracted from the matrix with 5 % formic acid and ACN, and speed vac dried for further analysis by LC–MS.

LC–MS/MS was performed using a system of liquid chromatography attached to a mass spectrometer (Cap-LC-nano-ESI-Q-TOF, Micromass-Waters). The dried peptides were re-suspended in Formic acid 1 %, and 4–10 µl were injected into C18 reverse phase capillarity column (75 μm Ø, 3 μm particle, 15 cm L, Nanoase Atlantis, Waters). Applied gradient was 5–60 % B in 35 min (A: 2 % ACN, 0.1 % FA; B: 90 % ACN, 0.1 %FA).

Eluted peptides were ionized with electrospray (ES) (emitter nano-ES PicoTip™, New Objective) applying a voltage of 2 kV to the capillary and 60 V to the cone.

A range of mass peptides 400–1800 m/z was analyzed in full-scan MS mode (scan time 1 min) with a resolution of 10000 FWHM. In this range, the 10 most abundant peptides were selected for CID fragmentation (collision-induced dissociation; collision energy 20 eV; Argon gas) in the MS/MS analysis (scan time 1 s, range 100–1700 m/z). As a result, a PKL file of peptide fragmentation was obtained to search for and match sequences on databases.

Protein identification was performed by searching for the combined MS and MS/MS spectra against the green plant NCBI database using a local MASCOT search engine (V.1.9) on a GPS (V. 3.5, ABI) server. Search parameters were set as follows:

- Database/Taxonomy: NCBInr all and ESTs (Expressed Sequenced Tags) plants.
- Enzyme: trypsin.
- Missed cleavage: 2.
- Fixed modifications : carbamidomethyl of cystein.
- Variable modifications: oxidation of methionine and pyro-Glue (N-term Glutamine).
- Peptide tolerance: 100 ppm and 0.25 Da (respectively, for MS and MS/MS spectra).

Proteins containing at least two peptides with confidence interval (CI) values no less than 95 % were considered as being identified.

### Quantitative determination of protein carbonylation

The quantitative measurement of carbonyl groups was performed using the aliquot for each sampling point corresponding to 50 ng of protein, previously extracted and quantified as described (Levine et al. 1994; Juszczuk et al. 2008) with minor modifications. Briefly, 750 µl of 10 mM DNPH in 2 M HCl was added to a volume of protein extract, corresponding to 60 µg of protein, and incubated in the dark for 1 h being vigorously mixed every 10 min. The labeling was stopped by adding 0.5 ml of 20 % trichloroacetic acid (TCA) and left on ice for 10 min. After centrifugation for 5 min at 10,000*g* the pellet was washed three times with 2.5 ml of ethanol:ethyl acetate (1/1, v/v). After each washing step, the samples were centrifuged for 5 min at 10,000*g*. The final pellet was dissolved in 1 ml of 6 M guanidine hydrochloride (50950, Fluka) with 0.5 M potassium phosphate, pH 2.5, incubated at room temperature for 10 min and centrifuged for 5 min at 10,000*g*. The absorbance of the supernatant was measured at 375 nm, and the concentration of carbonyls was calculated from the molar extinction coefficient for DNPH, ε375 = 22.000 M^−1^ cm^−1^.

### Determination of ABA, ABA-GE, PA, DPA and JA

Leaf content of abscisic acid (ABA), abscisic acid glucose ester (ABA-GE), phaseic acid (PA), dehydrophaseic (DPA) and jasmonic acid (JA) were simultaneously analyzed by HPLC MS/MS as previously described by (López-Carbonell et al. 2009; Brossa et al. 2011). Standards of PA, DPA and 7′-hydroxy ABA and their deuterated forms were purchased at Plant Biotechnology Institute, National Research Council, Saskatoon (Canada). Their transitions and MS settings were determined and appropriate calibration curves were performed.

Briefly, 100 mg of leaves was ground in liquid nitrogen with a mortar and pestle and extracted with 759 µl of methanol–water–acetic acid (90:9:1 by vol.). Deuterium-labeled internal standards were added to each of the samples and replicates at the beginning of the extraction procedure. Extracts were vortexed and incubated under ultrasonication and centrifuged. The supernatants were collected and the pellets were re-extracted. Afterwards, 5 µl of each sample was injected into the LC system (Acquity UPLC, Waters) using a Waters X-Bridge C18 column. Quantification was performed using MS/MS on an API 3000™ triple quadruple mass spectrometer.

### Analyses of reduced and oxidized forms of glutathione and ascorbate

Ascorbate and glutathione (reduced and oxidized forms) were determined by a plate-reader method (Queval and Noctor 2007) with slight modifications. Briefly, ten leaves were ground in liquid nitrogen with a mortar and pestle. 100 mg was weighed followed by the addition of 1 ml of extraction buffer (6 % meta-phosphoric acid, 2.5 mM EDTA) and clarified with 10 min centrifugation at 8,000*g*. Finally, the extracts were neutralized and diluted before spectrophotometric readings on a 96-well quartz microplate (Hellma Hispania SL, Badalona, Spain). The levels of ascorbate (AsA) (reduced) and dehydroascorbate (DHA) (oxidized) were determined using ascorbate oxidase (AO) and DTT (Foyer et al. 1983). AO specifically oxidizes all reduced ascorbate in the sample. Thus, the decrease in OD at 265 nm is related to AsA concentration. Alternatively, when the samples are incubated with DTT, DHA is reduced to AsA and the increase in OD is proportional to the initial DHA concentration.

From the same clarified extracts used for AsA determination, reduced and oxidized glutathione forms were measured by the glutathione reductase (GR) recycling assay (Tietze 1969); while for oxidized glutathione, the sample was prior incubated with 2-vinylpyridine (Griffith 1980). When NADPH is present in sample, glutathione is interconverted into oxidized and reduced forms by GR; this cycle causes that di-thio-nitro-benzoic acid (DTNB) is ruptured increasing the absorbance at 412 nm. The increase in OD is monitorized during a exact period of time (e.g., 5 min) and it should be positively correlated with glutathione concentration.

### Statistical analyses

The data were analyzed using PASW for Windows v.17.0.2 (SPSS Inc., Chicago IL). ANOVA was used to compare mean values at a range of sampling times for all determined parameters and measurements. The post hoc Duncan’s test was applied. Significance levels of 95 % (*P* < 0.05) are indicated in figure legends. At each sampling time, significantly different means are marked with different letters.

## Results

### Plant responses to water stress and recovery: water status and leaf mass area

**Fig. 2:**
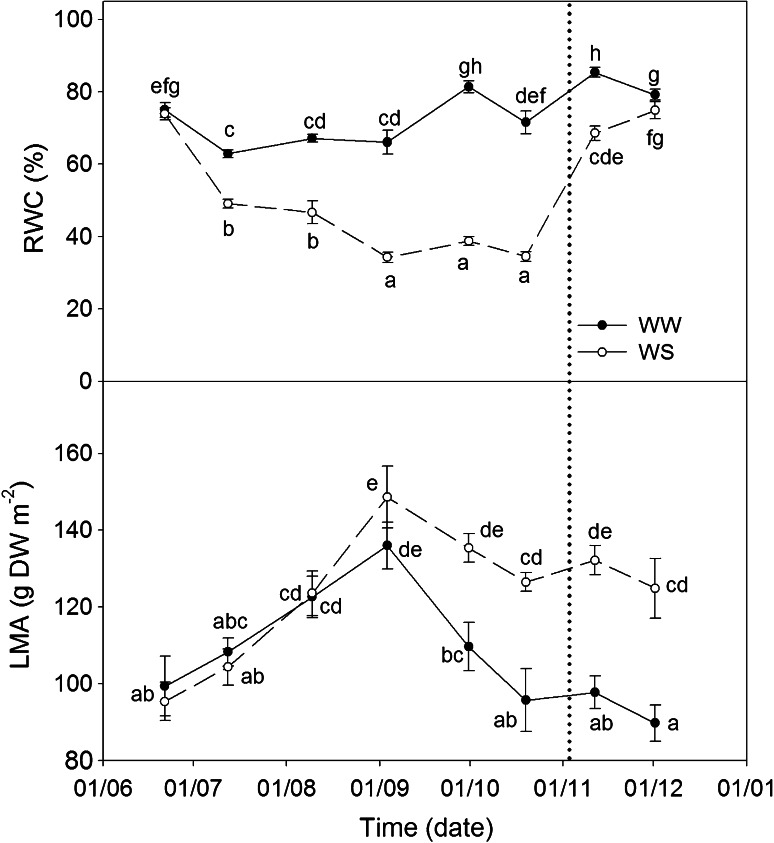
Time-course of plant water status and leaf mass per area in WW and WS *C. albidus* plants. Relative water content (RWC %), and Leaf Mass per Area (LMA) in leaves of well-watered (*closed circle*, WW) and water-stressed (*open circle*, WS) plants. For the experiment, leaves situated from 5 to 15 cm from the apex were collected at midday (at maximum diurnal incident PPFD) on clear sunny days, once every month. A watering recovery of WS plants was performed on November 5. Values with *different letters* indicate significant differences at *P* < 0.05 %. Data are mean ± SE. At least 10 individuals were used in each sampling point

At the beginning of the experiment, RWC of plants from both plots (WW and WS plants) was c.a. 75 %. Variations in the RWC of WW plants were observed throughout the experiment but it was always significantly higher than in WS plants (Fig. 2a). In WS plants, 3 weeks after the beginning of water stress (August 9), RWC decreased to 49 % and kept on decreasing progressively, reaching the lowest value (33 %) at the beginning of September. This value remained constant until the recovery period started. On November 11, 6 days after the water recovery started, plants showed a remarkable increase in their RWC (up to 68 %) but still significantly lower than WW values. Finally, by December 1, the RWC in WW and WS plants showed no significant differences, with values of 75 and 79 %, respectively. Leaf mass per area (LMA) (Fig. 2b) values increased in both treatments during summer (until September 3) as result of an increase in leaf dry weight (data no shown). At the end of September, WW plants showed a sharp decrease in LMA caused by the growth of new leaves. Meanwhile, WS plants maintained their LMA throughout autumn, even after recovery.

In this study, we examined the dynamics of the leaf proteome by simultaneously measuring the following parameters: gas exchange and stress markers such as maximum efficiency of photosystem II (PSII) photochemistry (*F*_v_/*F*_m_) and protein carbonylation; antioxidants such as ascorbate and glutathione; and hormones such as ABA and its metabolites, ABA-GE, PA, DPA and jasmonic acid. The aim was to shed new light on the mechanisms of plant resistance to drought followed by water recovery in *C. albidus* plants growing under natural climatic conditions.

### Photosynthesis

**Fig. 3:**
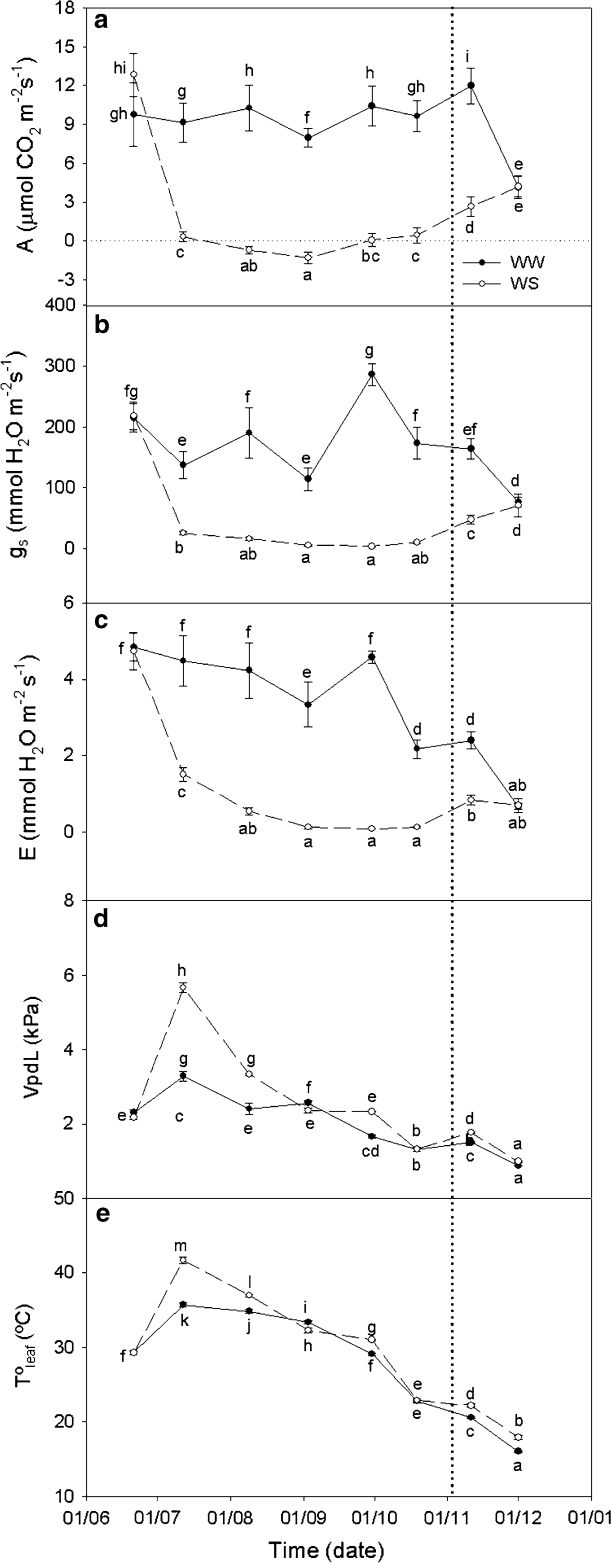
Gas exchange measurements in WW and WS *C. albidus* plants. **a** Net assimilation rate (A, μmol m^−2^ s^−1^). **b** Stomatal conductance (g_s_, mmol m^−2^ s^−1^). **c** Transpiration rate (E, mmol m^−2^ s^−1^). **d** Leaf-to-air vapor pressure deficit (VpdL, kPa). **e** Leaf temperature (Tº_leaf_, °C) in leaves of well-watered (*closed circle*, WW) and water-stressed (*open circle*, WS) plants during samplings performed between June 21 and December 1, 2010. A watering recovery of WS plants was performed on November 5. Values with *different letters* indicate significant differences at *P* < 0.05 %. Data are mean ± SE. At least 10 individuals were used in each sampling point

Net photosynthetic rates, measured at midday (Fig. 3a), ranged from 9 to 12 µmol m^−2 ^s^−1^ in WW plants from the beginning of the experiment until November when photosynthesis began decreasing in parallel with the decrease in both the maximum and minimum *T*_air_ and *T*_leaf_ (Fig. 3d). However, in WS plants, a significant decrease in CO_2_ assimilation (to 0 µmol m^−2^ s^−1^) was observed after 3 weeks of water deprivation, concomitant with a significant decrease in RWC and an increase in VPD (Fig. 3e). Values of net photosynthesis measured at midday in WS plants remained at the compensation point for CO_2_ during the drought period, increasing progressively after recovery and reaching values of 5 µmol m^−2^ s^−1^ as in WW plants.

In WW plants, g_s_ values always remained over 100 mmol H_2_O m^−2^ s^−1^; while in WS plants, g_s_ values drastically decreased to below 50 mmol H_2_O m^−2^ s^−1^ when the water stress treatment started (Fig. 3b). Low *g*_s_ values in WS plants minimized the water loss, as shown by low E values (Fig. 3c) and limited photosynthesis rates (Fig. 3a). Finally, WS plants reached WW assimilation rates 1 month after water recovery conditions started, on December 1. The above results are typical for more drought-tolerant plants confirming that *C. albidus* has a resistance mechanism to drought.

### Oxidative markers: *F*_v_/*F*_m_ and protein carbonylation

**Fig. 4:**
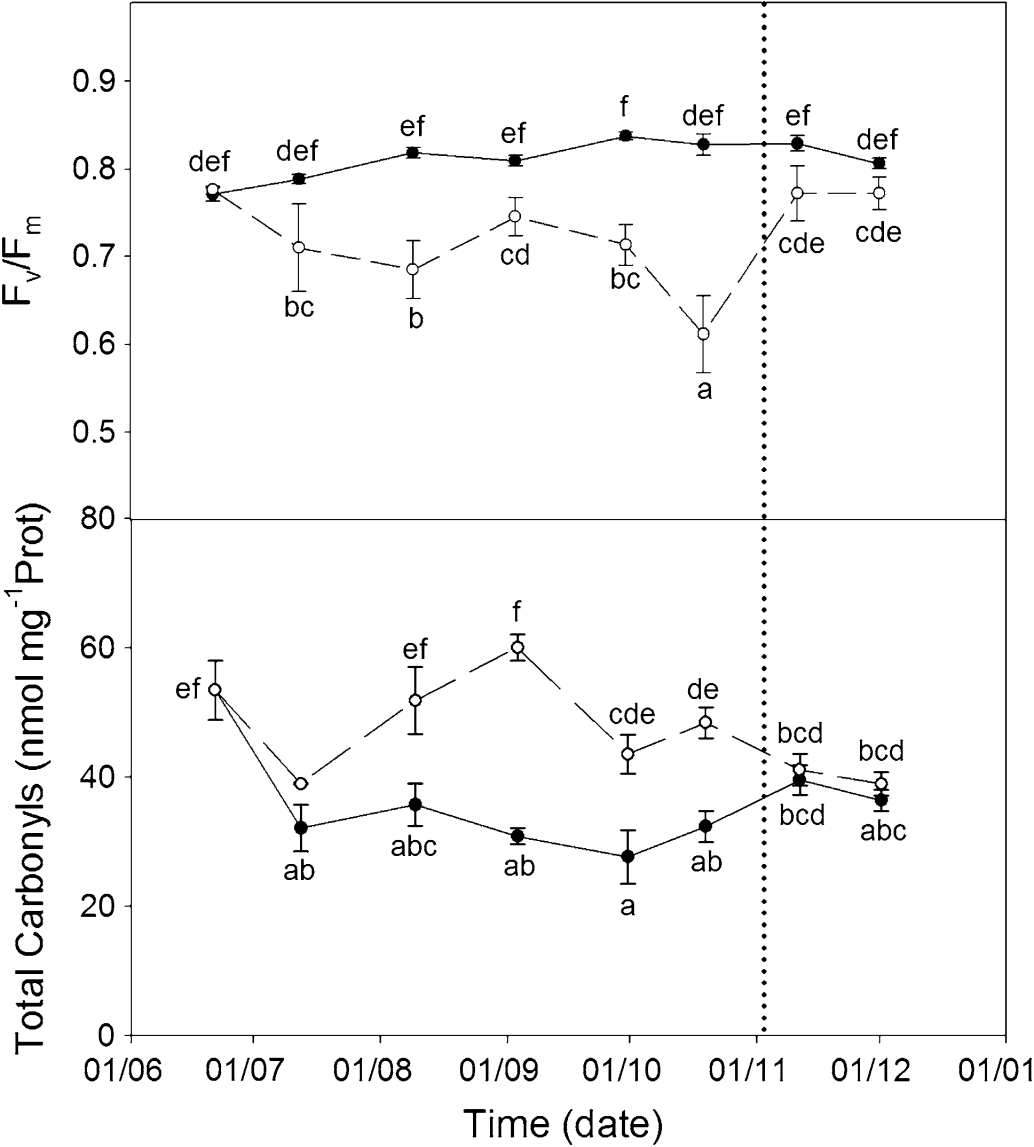
Time-course of oxidative stress markers: *F*_v_/*F*_m_ and protein carbonylation in WW and WS *C. albidus* plants.Variations on maximum efficiency of PSII (*F*_v_/*F*_m_) and protein carbonylation (nmol mg^−1^ Prot) in leaves of well-watered (*closed circle*, WW) and water-stressed (*open circle*, WS) plants. For the experiment, leaves situated at 5–15 cm from the apex were collected at midday (at maximum diurnal incident PPFD) on clear sunny days, once every month. Watering recovery of WS plants was performed on November 5. Values with *different letters* indicate significant differences at *P* < 0.05 %. Data are mean ± SE. At least 10 individuals were used in each sampling point

The maximum efficiency of PSII photochemistry (F_v_/F_m_ ratio; Fig. 4) showed the highest values in WW treatment (maximum of 0.84), and varied throughout the time-course of the experiment, concomitant with the decrease in accumulated radiation. In WS plants, the *F*_v_/*F*_m_ ratio decreased to 0.71 after 3 weeks of withholding water, then levels remained lower compared to WW, reaching a minimum on October 20 (0.61) at maximum water stress. After water recovery, *F*_v_/*F*_m_ values in WS plants returned to initial values (0.77); nevertheless, recovered plants remained slightly downregulated compared to WW (Fig. 4).

Protein oxidation by carbonylation (Fig. 4) was slightly but significantly higher in WS plants with respect to WW plants (40–60 nmol mg^−1^ protein, 32–39 nmol mg^−1^ protein, respectively) and decreased slightly after recovery reaching the same values as those of WW plants (39 nmol mg^−1^ protein). Thus, even with higher levels and variations in protein carbonylation shown in WS plants, drought has little effect on total protein oxidation in *C. albidus* plants.

### Protein identification

Analysis of 2-DE gels allowed for the identification of distinct protein expression patterns in WW and WS treatments. All obtained spot maps (Fig. S1, Suppl. Material) were statistically analyzed and then separated into three groups: WW, WS treatment and recovery period, respectively. In total, 259 spots were assigned as reproducible in the 32 analyzed gels. Replicate images of every sampling point were grouped and statistical analysis (*P* < 0.05) revealed that 70 spots out of the 259 presented were differentially expressed. These protein spots were selected and excised from gels for further identification. From the 70 selected spots, 53 had a positive identification in the BLAST and/or EST databases. The identified proteins are presented in Table 1. Some of the spots had a high score in the EST database with a previously described *Cistus creticus* trichome library (Falara et al. 2008). In these cases, the whole sequence of the matched clone from the CT library was compared to BLAST and an additional positive ID result was obtained (marked with * on Table 1).

**Table 1:** Proteins differentially expressed in *C. albidus* leaves of water-stressed plants, as identified by MALDI-TOF–MS/MS followed by a database search. no, spot number also according to Fig. 5

No	Putative id.	Acc. no.	Organism	NP.	SC (%)	Score	Measured		Theortical		Functional classification	Response	
							MW (kDa)	PI	MW (kDa)	PI		Max.	Min
Drought stress up-regulated proteins													
1	Cytosolic class I small heat shock protein type 2	gi|283482274	*R. oldhamii*	4	21	308	16.5	6.8	15.9	5.4	Stress response	S 3 Sept	WW 21 Jun
2	Lactoylglutathione lyase	gi|211906514	*G. hirsutum*	3	11	110	32.0	5.2	32.6	5.7	Stress response	S 20 Oct	WW 21 Jun
3	Heat shock protein	gi|159138935	*G. hirsutum*	1	5	48	19.0	5.4	18.0	6.2	Stress response	S 3 Sept	WW 21 Jun
4	Low molecular weight heat shock protein	gi|159138941	*G. hirsutum*	3	21	196	18.0	5.9	17.7	5.8	Stress response	S 3 Sept	WW 11 Nov
9	cp10-like protein	gi|21780187	*G. hirsutum*	1	3	51	27.0	5.3	26.8	7.8	Protein destination	S 3 Sept	WW 21 Jun
14	Dehydroascorbate reductase	gi|255564070	*R. communis*	3	17	97	23.5	6.1	23.8	5.9	Stress response	S 3 Sept	WW 11 Nov
15	Predicted similar to HSC70-1, ATP binding isoform 1	gi|225434994	*V. vinifera*	9	15	378	74.0	4.7	71.7	5.1	Stress response	S 3 Sept	WW 21 Jun
20	CII small heat shock protein 1	gi|259123935	*C. creticus* (*)	6	40	278	14.5	5.9	27.9	9.7	Stress response	S 3 Sept	WW 11 Nov
26	Superoxide dismutase [Cu–Zn]	gi|71040665	*A. hypogaea*	2	13	69	15.0	5.4	15.2	5.3	Stress response	S 3 Sept	WW 20 Oct
35	Heat shock protein	gi|224146037	*P. trichocarpa*	2	10	71	19.0	4.5	17.4	5.4	Stress response	S 3 Sept	SR 11 Nov
21	Phosphoglycerate kinase	gi|15219412	*A. thaliana*	8	19	376	41.0	5.5	42.2	5.5	Carbon metabolism	S 20 Oct	WW 9 Aug
32	Cytochrome b6-f complex iron-sulfur subunit, chloroplastic	gi|136707	*P. sativum* (**)	3	16	156	19.0	4.7	24.7	8.6	Energy	S 9 Aug	WW 20 Oct
33	Triosephosphate isomerase, cytosolic	gi|1351279	*Petunia x h* (**)	2	9	96	26.0	5.3	27.3	5.5	Energy	S 3 Sept	SR 11 Nov
5	Oxygen-evolving enhancer protein 2, chloroplastic	gi|8131593	*B. gymnorhiza* (**)	5	3	58	22.0	4.9	17.6	4.9	Photosynthesis	S 3 Sept	WW 11 Nov
18	Oxygen-evolving enhancer protein 2, chloroplastic	gi|350538909	*S. lycopersicum*	3	12	92	21.0	6.2	27.9	8.3	Photosynthesis	S 20 Oct	WW 11 Nov
23	Oxygen-evolving enhancer protein 3, chloroplastic	gi|11133850	*O. viciifolia* (**)	2	9	138	19.0	9.2	24.8	9.6	Photosynthesis	S 3 Sept	WW 20 Oct
23B	Lipid transfer protein precursor	gi|7012724	*G. hirsutum*	2	19	163	18.5	9.4	17.5	9.7	Cellular transport	S 3 Sept	WW 20 Oct
6	Non-specific lipid transfer protein PvLTP-24	gi|2347088	*P. vulgaris*	1	10	63	13.0	9.3	12.2	8.9	Cellular transport	S 3 Sept	WW 20 Oct
24	TPA: isoflavone reductase-like protein 5	gi|76559894	*C. creticus* (*)	2	10	229	33.0	5.6	28.7	9.5	Secondary metabolism	S 3 Sept	WW 20 Oct
Watering recovery up-regulated proteins													
25	Glycine dehydrogenase [decarboxylating] 1	gi|15225249	*A. thaliana*	5	4	159	125.0	6.2	114.7	6.2	Amino acid metabolism	SR 11 Nov	WW 11 Nov
27	Chloroplast photosystem I protein	gi|192764655	*P. tremula*	4	25	146	23.0	9.3	18.6	9.7	Photosynthesis	SR 11 Nov	WW 3 Sept
27B	Lipid transfer protein precursor	gi|7012724	*C. creticus* (*)	2	14	106	23.0	9.3	17.5	9.7	Cellular transport	SR 11 Nov	WW 3 Sept
29	Voltage-dependent anion-selective channel	gi|255558216	*R. communis*	1	4	63	28.0	9.2	29.4	7.9	Cellular transport	SR 11 Nov	WW 20 Oct
30	ATP synthase beta subunit	gi|4063550	*H. grandiflorum*	12	38	659	49.0	5.1	49.2	5.2	Energy	SR 11 Nov	S 20 Oct
31	Catalase isozyme 1	gi|1345674	*G. hirsutum*	2	5	84	55.0	6.6	57.3	6.6	Stress response	SR 11 Nov	S 3 Sept
Downregulated under water stress proteins													
42	Chloroplast sedoheptulose-1,7-bisphosphatase	gi|238563983	*S. lycopersicum*	7	14	441	43.0	4.9	43.0	6.2	Carbon metabolism	WW 11 Nov	S 9 Aug
52	Glyceraldehyde-3-phosphate dehydrogenase B subunit	gi|351726690	*Glycine max*	8	17	356	44.0	6.7	48.7	7.1	Carbon metabolism	WW 20 Oct	S 3 Sept
40	Glyceraldehyde-3-phosphate dehydrogenase	gi|300068297	*B. rapa*	5	16	154	42.0	6.4	42.8	8.5	Carbon metabolism	WW 20 Oct	S 20 Oct
55	Transketolase, chloroplastic	gi|68052991	*S. oleracea*	6	9	317	78.0	6.0	80.7	6.2	Carbon metabolism	WW 11 Nov	S 3 Sept
58	Transketolase, chloroplastic	gi|68052991	*S. oleracea*	4	6	187	78.0	6.1	80.7	6.2	Carbon metabolism	WW 11 Nov	S 20 Oct
60	Transketolase, chloroplastic	gi|68052991	*S. oleracea*	6	9	337	78.0	6.1	80.7	6.2	Carbon metabolism	WW 11 Nov	S 3 Sept
61	Phosphoribulose kinase	gi|117296238	*Acer saccharum*	4	21	255	37.0	5.3	22.8	4.8	Carbon metabolism	WW 11 Nov	S 9 Aug
65	Glyceraldehyde 3-phosphate dehydrogenase, putative	gi|255539282	*R. communis*	4	10	200	44.0	7.0	49.1	7.6	Carbon metabolism	WW 11 Nov	S 3 Sept
70	Rubisco large subunit	gi|2654331	*Cistus x revolii*	14	20	591	52.0	6.3	51.4	6.1	Carbon metabolism	WW 21 Jun	S 20 Oct
28	Rubisco large subunit	gi|209418680	*Cistus albidus*	7	12	266	52.0	9.2	51.7	6.0	Carbon metabolism	WW 3 Sept	S 20 Oct
48	Rubisco large subunit	gi|209418710	*C. populifolius*	10	18	603	48.0	4.4	51.7	6.3	Carbon metabolism	WW 3 Sept	S 3 Sept
43	Ribulose bisphosphate carboxylase small chain	gi|15219826	*C. creticus*	10	36	449	8.0	4.0	27.3	9.3	Carbon metabolism	WW 9 Aug	S 9 Aug
64	Ribulose bisphosphate carboxylase	gi|16194	*A. thaliana*	2	4	52	7.0	6.6	20.6	7.6	Carbon metabolism	WW 9 Aug	S 20 Oct
53	Ribulose bisphosphate carboxylase small chain	gi|132080	*Nicotiana sylvestris*	2	6	108	7.0	6.6	20.7	6.6	Carbon metabolism	WW 3 Sept	S 20 Oct
67	Rubisco small subunit	gi|270383722	*E. globulus* (**)	9	12	111	13.0	9.3	23.2	10.0	Carbon metabolism	WW 9 Aug	S 20 Oct
46	Carbonic anhidrase	gi|8954289	*V. radiata*	3	13	151	29.0	6.0	35.8	7.6	Energy	WW 11 Nov	S 20 Oct
57	ATP synthase CF1 epsilon subunit	gi|91208908	*C. creticus* (*)	3	19	100	12.0	3.9	19.1	4.7	Energy	WW 20 Oct	S 9 Aug
59	Carbonic anhydrase, chloroplastic	gi|115472	*S. oleracea*	5	15	287	28.0	6.3	34.9	6.6	Energy	WW 9 Aug	S 20 Oct
66	ATP synthase CF1 alpha subunit	gi|91208887	*G. hirsutum*	11	23	571	50.0	6.1	55.4	5.3	Energy	WW 9 Aug	S 20 Oct
69	ATP synthase CF1 alpha subunit	gi|91208887	*G. hirsutum*	2	4	109	8.0	4.9	55.4	5.3	Energy	WW 9 Aug	S 20 Oct
56B	Oxygen-evolving enhancer protein 2, chloroplastic	gi|11134035	*S. tuberosum*	5	15	193	17.0	4.8	28.2	8.3	Photosynthesis	WW 9 Aug	S 9 Aug
63	Chloroplast oxygen-evolving enhancer protein 1	gi|346230039	*D. longan*	11	33	642	33.0	4.9	35.4	5.8	Photosynthesis	WW 9 Aug	S 9 Aug
68	Light-harvesting complex I protein Lhca3	gi|224131950	*P. trichocarpa*	1	6	91	24.0	6.2	29.6	9.1	Photosynthesis	WW 11 Nov	S 9 Aug
47	Rubisco activase alpha 2	gi|78100212	*G. hirsutum*	13	18	550	44.0	4.8	46.9	4.8	Protein binding	WW 3 Sept	S 20 Oct
41	Catalytic/coenzyme binding protein	gi|297843724	*A. lyrata*	6	19	171	42.0	6.5	42.6	8.5	Protein binding	WW 3 Sept	S 20 Oct
62	Unknown (putative binding protein)	gi|217073920	*M. truncatula*	3	10	123	35.0	5.3	37.6	6.3	Protein binding	WW 9 Aug	S 9 Aug
41B	Unknown (putative NAD(P) binding site)	gi|255647108	*G. max*	6	14	216	42.0	6.5	42.1	7.7	Nucleotide binding	WW 3 Sept	S 9 Aug
45	GTP-binding nuclear protein Ran/TC4	gi|585783	*V. fava*	16	41	610	26.0	6.8	25.6	6.4	Nucleotide binding	WW 9 Aug	S 9 Aug
56	Histone H2B	gi|7387726	*G. hirsutum*	4	22	149	16.0	4.8	16.1	10.1	Cell cycle	WW 9 Aug	S 9 Aug
49	Hypothetical protein SORBIDRAFT_04g033580	gi|242066558	*S. bicolor*	3	11	146	16.0	4.6	28.7	8.8	Others	WW 21 Jun	S 9 Aug
50	Plastidic aldolase	gi|4827251	*N. paniculata* (**)	3	6	195	36.0	4.9	42.8	6.9	Carbon metabolism	WW 11 Nov	S 9 Aug

Acc. no., NCBI protein database accession number. Organism, species with the highest homology. In the cases where a protein was identified based on the EST database (*) (*Cistus creticus* library) the EST sequence was blasted and the protein with the highest and significant homology was given. Some proteins were identified on both databases (**)

*NP* number of matched peptides, *SC* sequence coverage

To simplify protein data interpretation, three groups were organized based on the following criteria: the first group consisted of 35 spots that were found to be generally more abundant in samples from the WS treatment on the days August 9, September 3 and October 20 (upregulated); and the second group consisted of four spots whose expression was significantly higher in the stress recovery sampling point on November 11 in both WW and WS plants. Finally, the third group containing 31 proteins was significantly less abundant or absent in the WS treatment (downregulated).

A total of 17 proteins were unidentified, 13 of which were upregulated under WS treatment while 4 were downregulated under WS. Out of the 17, only 7 displayed non-significant hits on the mascot search on either the NCBI or EST databases. For the remaining 10 protein spots, several peptides with non-correspondence on databases were observed. Furthermore, for 4 spots (# 23, #27, #41 and #56), two positive IDs were obtained. We were also able to observe several forms or fragments of the same protein for spots #70, #28 and #48, all corresponding to the ribulose-1 5-bisphosphate carboxylase oxygenase (RuBisCO) large subunit. Their experimental Mr was similar to the theoretical but they clearly differed in their pI. This suggests that in the case of spots #48 and #28, protein modifications or partial degradation may have occurred; whereas spot #70 may correspond to intact RuBisCO, since it is by far the most intense protein spot in all gels. In the case of the RuBisCO small subunit, there were four spots which corresponded to this ID (#43, #53, #64 and #67). However, according to experimental data none of them correspond to the fully intact small subunit.

Looking at the functional classification of the identified proteins, the majority of these were involved in C-compound and carbohydrate metabolism. Within these, only phosphoglycerate kinase (spot #21) appeared once as upregulated under WS conditions, while the other 14 spots were all downregulated under WS. Apart from spots corresponding to RuBisCO fragments, 3 transketolases (spots #55, #58 and #60), 2 forms of glyceraldehyde-3-phosphate dehydrogenase (spots #40 and #53), a chloroplast sedoheptulose-1 7-bisphosphatase (spot #42) and a phosphoribulose kinase (spot #94) were significantly more expressed in the WW treatment than in WS.

The second most representative category of identified proteins was classified as energy related (excluding the photosynthesis sub-group). Spots #57, #66 and #69 correspond to ATP syntase subunits and were found in the downregulated under WS group, as well as two spots identified as carbonic anhydrase (#46 and #59). In contrast, a triosephosphate isomerase (spot #33) and the iron-sulfur subunit of the chloroplastic cytochrome b-6 (spot #32) were found to be upregulated under WS treatment.

Within the photosynthesis sub-group, oxygen-evolving enhancer proteins (spots #5, #18, #23, #56B and #63) were either found to be up- or downregulated under WS. RuBisCO activase (spot #47) as well as other binding proteins and cofactors (spots #41, #45, #56 and #62) were more expressed in the WW treatment.

**Fig. 5:**
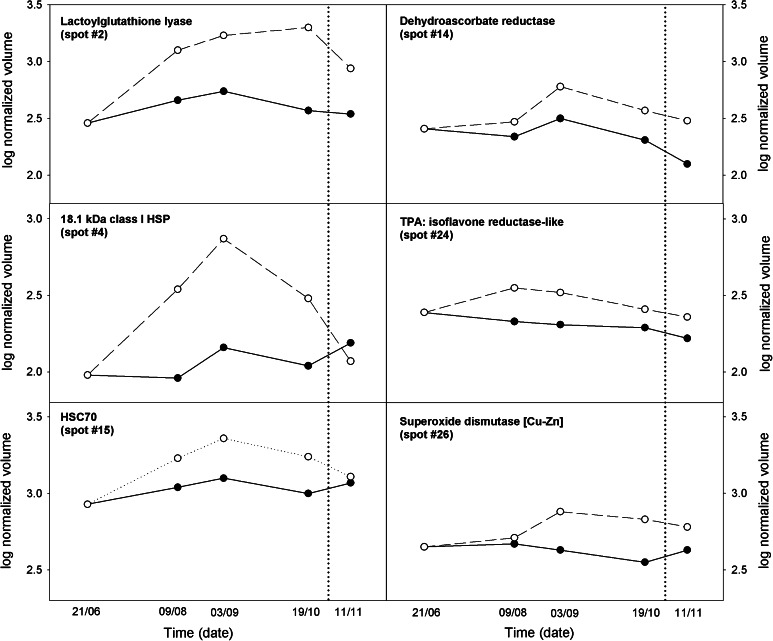
Dynamics of protein identification in water-stressed *C. albidus* plants. Log normalized spot volume during time-course of the experiment of six up-regulated spots on WS plants

Several stress-responsive proteins were identified as being up-regulated proteins under WS (Fig. 5) for instance: six heat shock proteins (spots #1,#3,#4,#15,#20 and #35), lactoylglutathione lyase (spot #2),dehydroascorbate reductase (spot #14), isoflavone reductase-like (spot·#24) and superoxide dismutase Cu–Zn (#26).

### Antioxidant response

**Fig. 6:**
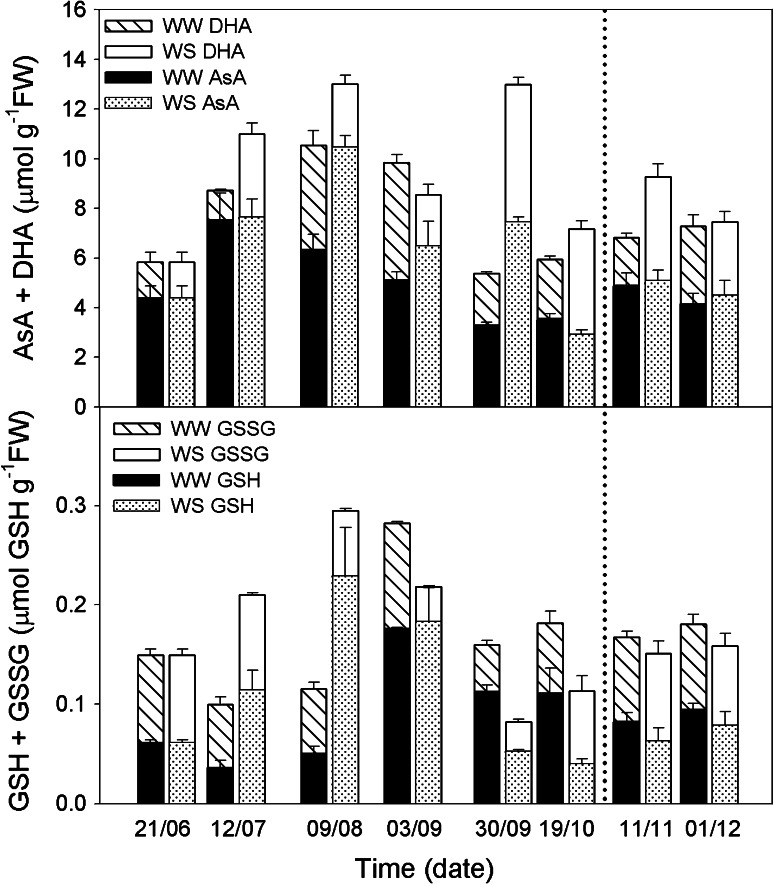
Leaf content of ascorbate and glutathione oxidized and reduced forms. Variations in total ascorbate (AA + DHA, µmol g^−1^FW; *upper graph*). Total glutathione (GSH + GSSG, µmol GSH g^−1^FW) in leaves of well-watered (*closed circle*, WW) and water-stressed (*open circle*, WS) *C. albidus* plants (*lower graph*). A watering recovery of WS plants was performed on November 5. Values with *different letters* indicate significant differences at *P* < 0.05 %. Data are mean ± SE. At least 10 individuals were used in each sampling point

Given that drought induced oxidative stress (Fig. 4), experiments were carried out to examine whether variations in total ascorbate (AsA + DHA) content and glutathione total content (GSH + GSSG) were related to stress parameters in WS plants. In water-stressed plants, total ascorbate and glutathione increased about 2.2-fold and 3-fold, respectively, from June to August 9. Both antioxidant molecules reached the maximum concentration at the beginning of August (12 µmol g^−1^ FW for total ascorbate and 0.3 µmol g^−1^ FW for total glutathione) (Fig. 6) A new peak of total ascorbate was observed at the beginning of November. There is a difference between the two peaks observed for total ascorbate where the first peak observed in August indicates an increase in AsA, while the second peak observed in November indicates an increase in DHA (Fig. 6).

### ABA metabolites and JA content

**Fig. 7:**
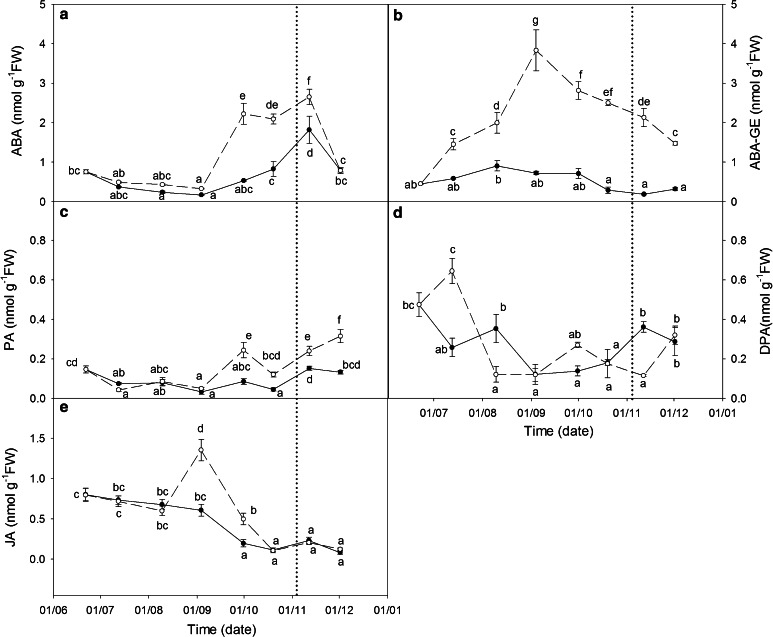
Endogenous concentration of ABA (**a**), ABA-GE (**b**), PA (**c**), DPA (**d**) and JA (**e**) (nmol g^−1^ FW) in leaves of well-watered (*closed circle*, WW) and water-stressed (*open circle*, WS) *C. albidus* plants. A watering recovery of WS plants was performed on November 5. Values with *different letters* indicate significant differences at *P* < 0.05 %. Data are mean ± SE. At least 10 individuals were used in each sampling point

To obtain an integrated view of stress signaling in *C. albidus*, HPLC–MS/MS analyses of leaf endogenous ABA metabolites and JA content were performed. On the one hand, ABA, ABA-GE and intermediate metabolites (PA and DPA) data revealed differences in ABA turnover between both treatments (Fig. 7). From August 9 until recovery on November 11, free ABA was always significantly higher in WS treatment when the data was compared separately for each sampling point (performing a T-student with 0.05 significance, data not shown). However, when all data points were compared, a notable ABA peak was only observed after September 30. By November 11, a late ABA peak was detected in WW plants, and by December 1, ABA levels in both treatments were similar.

ABA-GE remained below 1 nmol g^−1^ FW in WW plants, while under the WS treatment it rose notably on July 12, and kept on increasing until reaching a maximum at the September 3, sampling point (3.83 nmol g^−1^ FW). Thereafter, we observed a decrease of ABA-GE in WS plants but this never reached the low values observed in the WW treatment.

PA and DPA express the rate of ABA catabolism. PA followed a similar pattern to that observed in ABA accumulation but at much lower concentrations. In WS plants, PA concentration increased during the recovery period reaching a maximum on December 1 (0.32 nmol g^−1^ FW). DPA content in WW plants was 0.47 nmol g^−1^ FW on June 21 and from that day onwards DPA levels followed the ABA pattern with a peak on November 11. In contrast, DPA under WS conditions increased initially at the beginning of the stress treatment, and from this point onwards dropped to very low values (0.12 nmol g^−1^ FW) not following the ABA trend. 7′-hydroxy-ABA was not detected in any of the samples.

JA content decreased progressively in WW plants during the experiment, while in WS plants a peak was observed on September 3 (1.35 nmol g^−1^ FW). Following this maximum, JA levels decreased to values similar to those recorded for WW plants.

## Discussion

In the present study, we used an integrated proteomic approach, plant hormones and plant physiological processes to unravel the mechanisms underlying mediated drought stress resistance in *C. albidus*.

Proteomic analysis revealed that the abundance of proteins from several molecular processes was affected by drought stress. However, to date, there is a lack of comprehensive proteomic analyses for Mediterranean plants subjected to drought followed by recovery (Pinheiro et al. 2014).

The list of identified proteins in *C. albidus* showed low scores because of the lack of information about *Cistus* sp., however, several matches were found in *Gossypium hirsutum* (cotton plant) which belongs to the same order (Malvales) as *Cistus* sp. Furthermore, the ATPase β subunit that was identified and which was found to be upregulated during recovery (spot # 30) in *Helianthemum grandiflorum* belongs to the Cistaceae family.

### Redox proteomics and phytohormone interactions

Drought is considered to cause oxidative stress (Noctor et al. 2014) but the effects of drought on oxidative stress are less evident than the effects of some other stresses (e.g., many biotic stresses). Decreased water availability may induce progressive rather than acute oxidative stress and a myriad of molecular and physiological processes are needed to control the dynamics of this progressive stress. Among the mechanisms that control the dynamics of drought-oxidative stress, a close relationship between oxidative stress, enzymatic as well as low molecular antioxidants and phytohormones, such as ABA and its metabolites and JA, is essential. Common effects upon the perception of environmental stress include the differential expression of the plant proteome and the synthesis of novel regulatory proteins for acclimation to stress conditions. The effects of drought on the expression, abundance and activities of the major antioxidant enzymes (e.g., SOD, CAT, APX, DHAR, Gly1) have been reported (Han et al. 2012) suggesting that an antioxidant system is induced to protect plants from damage during drought stress.

The effect of ABA on the modulation of plant responses to drought is well known (Nambara and Marion-Poll 2005). One mode of ABA enhanced water stress resistance is associated with the induction of antioxidant defense systems (Miller et al. 2010; Ye et al. 2011), including enzymatic and non-enzymatic antioxidants, which protect plants cells against oxidative damage. It has been shown that ABA induces upregulation in the activities SOD, Gly 1 and APX. However, the mechanisms of how ABA regulates the antioxidant defense remain to be determined. Our results showed a plausible interaction between leaf proteome variation and phytohormones in plants subjected to stress following recovery.

The redox enzymes dehydroascorbate reductase (#14), superoxide dismutase (#26) and lactoylglutathione lyase (Gly1 (#2)) were upregulated in *C. albidus* leaves where Gly1 was the first to increase and also had the greatest increase at the beginning of stress. Moreover, it showed significant differences in relation to control plants. The accumulation of these enzymes indicates that ROS scavenging mechanisms were activated as a response to drought stress in *C. albidus* leaves (Fig. 5). Gly1 is described as a key enzyme in methylglyoxal detoxification (Dixon et al. 2005). Methylglyoxal is a cytotoxic compound produced in many cellular metabolic processes that accumulates in plants under diverse environmental stresses (Yadav et al. 2005). Its role in enhancing plant tolerance to stress has been widely assessed (Wu et al. 2013). Consequently, this result suggests that an effective response of *C. albidus* under stress conditions was activated.

An isoflavone reductase-like protein (#24) was overexpressed in the WS treatment, suggesting that in our experimental conditions, the metabolism of flavonoids was also affected by drought conditions and/or oxidative stress (Hernández et al. 2004).

In *C. albidus* shrubs, significant differences in ABA-GE accumulation were detected between treatments. WS plants revealed a higher accumulation of ABA-GE compared to WW plants and, moreover, ABA-GE accumulation preceded that of ABA in WS plants, suggesting that (1) ABA is rapidly linked to a receptor and used for plant responses to stress such as stomata closure and redox protein expression, and (2) ABA is conjugated rapidly with glucose leading to ABA-GE accumulation. Hydroxylation occurs only when WS is severe enough to need a higher free ABA concentration, as was detected on September 30: from this point ABA started to accumulate with a concomitant ABA-GE decrease. By October 20 and November 11, ABA slightly increases also in WW treatment, probably by the change of environmental conditions (lower temperatures, VPD and lower accumulated radiation) which may have also triggered a second release of free ABA in WS plants (November 11, peak). By the end of our experiment WW plants exhibited very low levels of ABA-GE while WS plants still contained a large amount, suggesting that differences in ABA metabolism remain between the two treatments several weeks after recovery. There is controversy regarding the activity of ABA-GE, as there are reports that suggest that ABA-GE is an active signal that can promote growth inhibition (Kato-Noguchi and Tanaka 2003). Furthermore, other reports support its importance in regulating the ABA pool through glucosidase activity (Arve et al. 2013).

Our results on *C. albidus* plants suggest that ABA is not accumulated at the beginning of stress, but rather most of the synthesized ABA is rapidly conjugated to ABA-GE for transport and storage to distribute the ABA signal throughout the plant, although it cannot be clarified, if ABA-GE is an active signal. The DPA content was maximal in WS plants on July 12, when ABA-GE started to accumulate under this treatment. PA and DPA also increased at the end of the experiment when high ABA levels were still present. These results are consistent with studies that reveal that ABA catabolism is a rapid process under stress (Ren et al. 2007), where either an increase in PA, conjugated PA, or in most cases DPA under drought follows an increase in ABA (Hansen and Dörffling 1999). On the contrary, catalase (#31) was also found to be upregulated in WS *C. albidus*, however, only on watering recovery sampling days. Catalase is the enzyme that catalyzes the decomposition of H_2_O_2_ to water and oxygen; and the accumulation of H_2_O_2_ in *C. albidus* leaves has been shown to be crucial to its acclimation to summer drought (Jubany-Marí et al. 2009).

Responses of catalase activity under drought stress are heterogeneous. CAT activity has been shown to increase and also to remain unchanged or even decrease under water stress (de Carvalho 2008). The regulation of CAT content and activity under drought is complex and it has been suggested that it is regulated by ABA, however, our study does not show any relation between CAT content and phytohormones. It has been suggested that CAT is a less susceptible scavenging enzyme than APX with regard to oxidative stress (de Carvalho 2008) and that CAT activity is only enhanced under severe drought stress whereas under moderate water stress H_2_O_2_ scavenging is carried out by APX. Our results suggest that the catalase expression profile is activated after the end of the drought stress period and is an indication that removal of accumulated H_2_O_2_ occurs only when the drought period is over, in order to balance oxidative stress signals.

As expected, stress-related proteins were found to be induced in leaves of *C. albidus* under WS treatment. One of these proteins, HSP70 (spot #15) was previously found to be upregulated under WS in drought-resistant plants (Akashi et al. 2011); HSP70 is involved in the refolding of non-native proteins, thus it prevents the aggregation of proteins and facilitates translocation processes (Wang et al. 2004) conferring drought resistance to plants.

Regarding the non-enzymatic antioxidant, ascorbate, it has also been reported that AsA concentration increases in leaves of *C. albidus* under WS conditions (Jubany-Marí et al. 2010b). In the present results, it has once again been shown that AsA has an important role in the survival/recovery of *C. albidus* plants, in coordination with the observed changes in stress protein expression. Interestingly, DHA remained low under WS concomitant with the higher expression of DHA reductase (Fig. 5) even though AsA content decreased on September 3. Furthermore, increases in DHA levels at the end of the WS period are paralleled by a decrease in DHA reductase.

Glutathione levels also increased under WS treatment, initially in parallel with an increase in AsA. In plants, glutathione actively participates in the regeneration of AsA from DHA in ROS detoxification processes (Asada 1999). Overall, the amount of GSH and GSSG forms should not necessarily always respond to the demands of the ascorbate redox state as it also has other functions under stress, namely it participates in the detoxifying processes of several metabolites (Marrs 1996) and in triggering protein glutathionylation (Dixon et al. 2005). The sharp decrease observed in total glutathione from September 3, could also be explained by its role in detoxifying processes because it occurred in parallel with the maximum Gly1 expression (Fig. 5b).

Increases in JA leaf content were observed in WS plants on September 3. This peak in JA levels could have contributed to the subsequent ABA accumulation. Recent studies suggest that JA levels have an influence on ABA accumulation, where this is involved in the crosstalk between ABA and JA essential to the coordination of drought and redox responses in *Arabidopsis* (Brossa et al. 2011). Similarly, WS in barley seedlings pre-treated with JA showed more than a fourfold accumulation in ABA when compared to WS barley seedlings that were not pre-treated with JA (Bandurska et al. 2003). Moreover, the role of JA in the regulation of ABA biosynthesis has been addressed (Kazan and Manners 2008).

Thus, our data suggest that ABA synthesis started early in *C. albidus* plants under WS treatment and the fact that we could not detect it as a free ABA form may be explained by the quick catabolism and glycosylation of ABA. Moreover, the JA peak observed on September 3 could have triggered the late free ABA accumulation through hydroxylation of the previously formed ABA-GE.

Focusing back on the role of JA in antioxidant regulation, our results also revealed that the JA peak in WS plants in September could be responsible for regulating the ascorbate redox state under WS in the period of maximum stress at the end of the summer. The influence of JA on ascorbate metabolism has been assessed in several studies and supports our data (Sasaki-Sekimoto et al. 2005; Wolucka et al. 2005; Piotrowska et al. 2010). Furthermore, the JA peak on September 3 also coincides with the decrease in GSH content and maximum Gly1 expression.

**Fig. 8:**
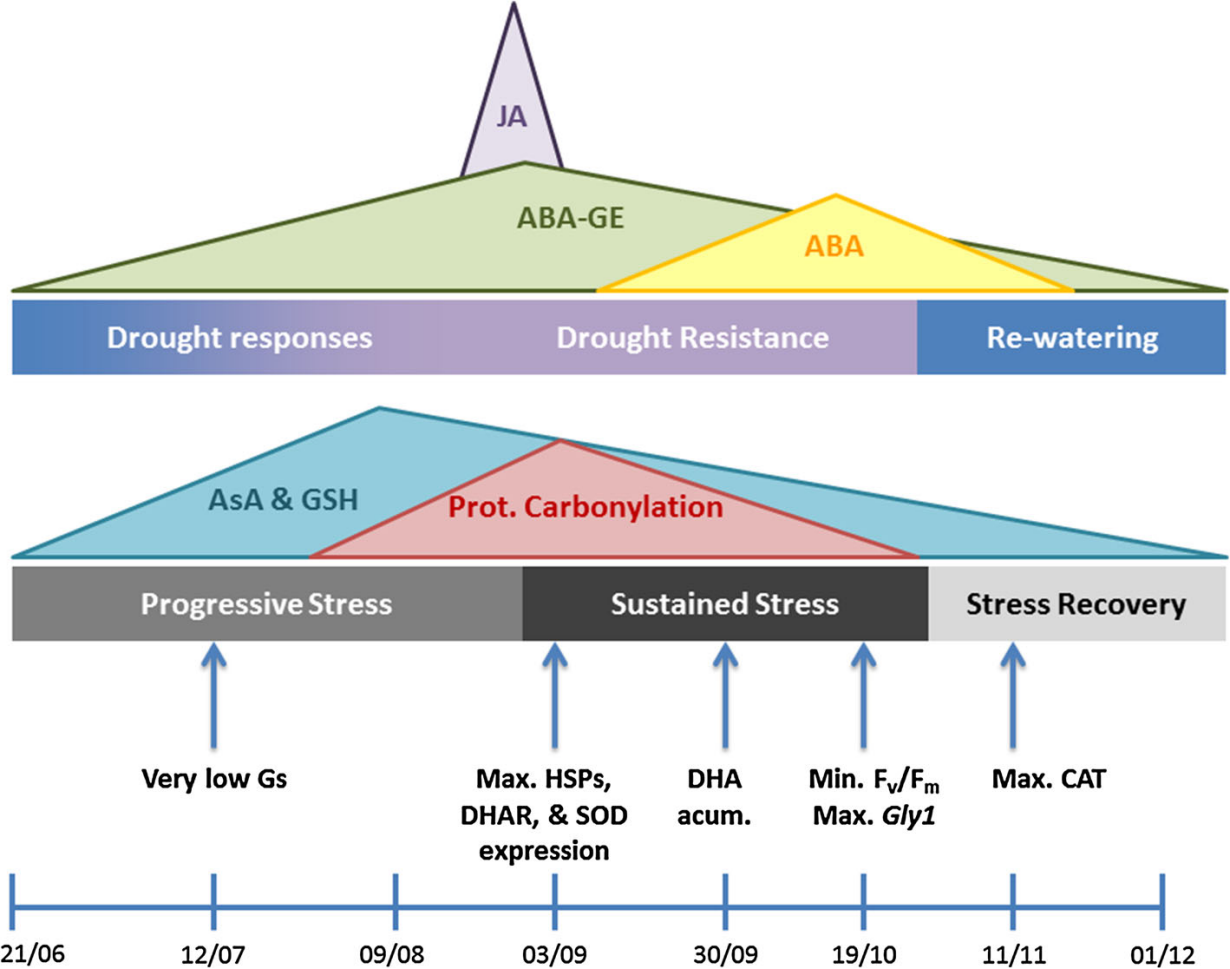
Time-course events during water deficit and oxidative stress progression in WS treatment. Summary of the most notable biochemical events in WS treatment determined by the evolution of hydration and oxidation. The illustrated periods were defined according to observed data. Dynamics of stress hormones, antioxidant and protein carbonylation are represented as peaks, while other significant data are indicated by *arrows*. *ABA* abscisic acid, *ABA-GE* abscisic acid glucose ester, *Acum*. accumulation, *AsA* ascorbic acid, *CAT* catalase, *DHA* dehydroascorbate, *DHAR* dehydroascorbate reductase, *F*_v_/*F*_m_ maximum efficiency of PSII, *Gs* stomatal conductance, *GSH* glutathione, *HSPs* Heat-shock proteins, *JA* jasmonic acid, *Prot.* protein, *SOD* superoxide dismutase

From these studies, we can conclude that *C. albidus* plants were subjected to a moderate water stress that produced a moderate oxidative stress and both were susceptible to recovery (Fig. 8). Plant hormones, ABA and its metabolites, and JA have a pivotal role in the modulation of antioxidant levels.

### Recovery of stress upon re-watering

*Cistus albidus* has the ability to remain alive in a dehydration state and then to resume normal growth after re-watering. Redox and detoxifying enzymes (Gly1, DHAR, SOD, CAT) did not return to initial levels after 11 days of re-watering; however, reduced forms of low molecular soluble antioxidants returned to initial levels rapidly, and only the removal of accumulated DHA was slower. This clearly explains why DHAR remains higher under WS recovery in comparison to WW treatment. Regarding the redox and detoxifying enzymes, the high expression of redox and detoxifying enzymes, after recovery on November 11, may also indicate that they may reduce oxidized components. Stress markers indicated that protein carbonlylation rapidly recovers WW levels while *F*_v_/*F*_m_ shows a slower trendline. In this case, both markers support that the sustained expression of the enzymes during recovery effectively removed oxidative damage. Besides, by November 11, most of the proteins involved in photosynthesis and carbon metabolism, such RubisCO small and large chains (#43, #48, #53, #70), transketolase and aldolase (#48, #50) chloroplastic oxygen evolving enhancer (#63) or light harvesting complex I/#63) or light harvesting complex I (#68), had recovered the same expression levels than WW plants (Fig. S2, Suppl. Material), what has been crucial for fast recovery of photosynthetic activity after drought. recovery.

### Comparative identification of proteins overall

To date, several studies assessing changes in the proteome under water deficit conditions have highlighted how a wide diversity of proteins related to photosynthesis and carbon metabolism are downregulated under water stress conditions e.g., RuBisCO activases, sedoheptulose biphosphate and β subunit of ATP synthases (Jorge et al. 2006; Sergeant et al. 2011). Overall, in our study, proteins involved in carbon metabolism were more expressed in plants under WW conditions as shown for spots #40, #28, #42, #43, #48, #52, #55, #58, #60, #61, #64, #65, #67B and #70. This is consistent with the carbon assimilation limitations that occurred under WS, which were also captured by gas exchange measurements. Furthermore, several studies have reported changes in carbon metabolism under drought conditions (Pinheiro et al. 2001; Chaves et al. 2003) and have shown the relocation of carbon reserves and also changes in the sucrose/starch ratio in leaves. Proteomic analyses have revealed that the abundance of proteins from several molecular processes is affected by drought stress. Under water deficit conditions *C. albidus* plants modify their protein expression to maintain the functionality of the most sensitive proteins in the electron transport and respiration rates. In this aspect, our results show similarities to other studies where energy and C-compound metabolism proteins were downregulated under stress (Bonhomme et al.2009a).

In summary, *C. albidus* plants represent a clear model of stress syndrome responses in plants followed by recovery. We can distinguish (after an alarm phase at the beginning of the experiment) an stress responses phase, from the beginning of July until the beginning of September characterized by a rapid decrease in plant water relations, gas exchange, closing stomata and decreasing A, E. and gs as well a decrease in *F*_v_/*F*_m_ and an increase in protein carbonylation (Fig. 8). Concomitant with this phase of stress responses, a stage of resistance started characterized by an increase in redox enzymes. Interestingly and referring to the levels of abscisic acid and its metabolites, mainly ABA-GE, an increase of this conjugated form appeared very soon on water-stressed plants and preceded the increase in free ABA. We suggest plausible explanations for the relationship ABA/ABA-GE in water-stressed plants. (1) The rapid closure of stomata does not allow the transport of ABA from roots to shoots, (2) ABA-GE values increase may be as a result of metabolic activity under stress and on changes in partitioning among compartments. (3) ABA-GE is likely to play a role in acclimation to stress since is still increasing through the period corresponding to absence of photosynthesis. So metabolic activity is going on and in this context antioxidative protein and photosynthesis proteins may play a role. More studies are needed to know the function of ABA-GE in drought-stressed plants. Once drought stress is removed plant resume growth, although ABA and ABA-GE and redox enzymes remain higher than in WW plants.

## Conclusion

*C. albidus* exposed to an extended summer drought gradually responded through an effective acclimation and managed to maintain a low RWC for several weeks. Photosynthesis was severely affected, as was observed under WS conditions through the clear downregulation of proteins involved in carbon metabolism and photosynthesis such as RuBisCO activases, sedoheptulose biphosphate and the β subunit of ATP synthases. Drought stress led to oxidative stress as indicated by the increase in protein carbonylation and the decrease in *F*_v_/*F*_m_. Oxidative protection was triggered by DHA reductase and stress-responsive detoxification enzymes such as lactoylglutathione lyase, catalase and superoxide dismutase and the high expression of several HSPs, all of which are involved in the ROS signaling network. Ascorbate and glutathione conferred the necessary oxidative protection regulated by JA, thus allowing plants to recover upon re-watering. These signals under WS were modulated by a large accumulation of ABA-GE, which simultaneously regulated an effective release of ABA. All observed responses lead to the conclusion that *C. albidus* has very efficient mechanisms of acclimation and protection in order to cope with long-term drought stress.

### **Author contribution statement**

RB, ML and LA conceived and designed research. RB and MP conducted field sampling, biochemical and data analysis. RF and MMC contributed with proteomic analysis. RB and LA wrote the manuscript. All authors read, reviewed and approved the manuscript.

## Electronic supplementary material

Below is the link to the electronic supplementary material.

Supplementary material 1 (PDF 1453 kb)

Supplementary material 2 (PDF 936 kb)

## Abbreviations

ABA: Abscisic acid
ABA-GE: β-D glucosyl ester
APX: Ascorbate peroxidase
AsA: Ascorbate
CAT: Catalase
DHA: Oxidized ascorbate
DPA: Dehydrophaseic
CAT: Catalase
GSH: Reduced glutathione
GSSFG: Oxidized glutathione
JA: Jasmonic acid
PA: Phaseic acid
ROS: Reactive oxygen species
SOD: Superoxide dismutase
WS: Water stressed
WW: Well watered
