# Supplementary figures and images for: Redox proteomics and physiological responses in Cistus albidus shrubs subjected to long-term summer drought followed by recovery
Source: Planta. 2014 Dec 13;241(4):803–22. doi: 10.1007/s00425-014-2221-0 (PMC4361772; doi:10.1007/s00425-014-2221-0)

**Fig. S1** Spots map of distinct expression patterns in WW and WS treatments

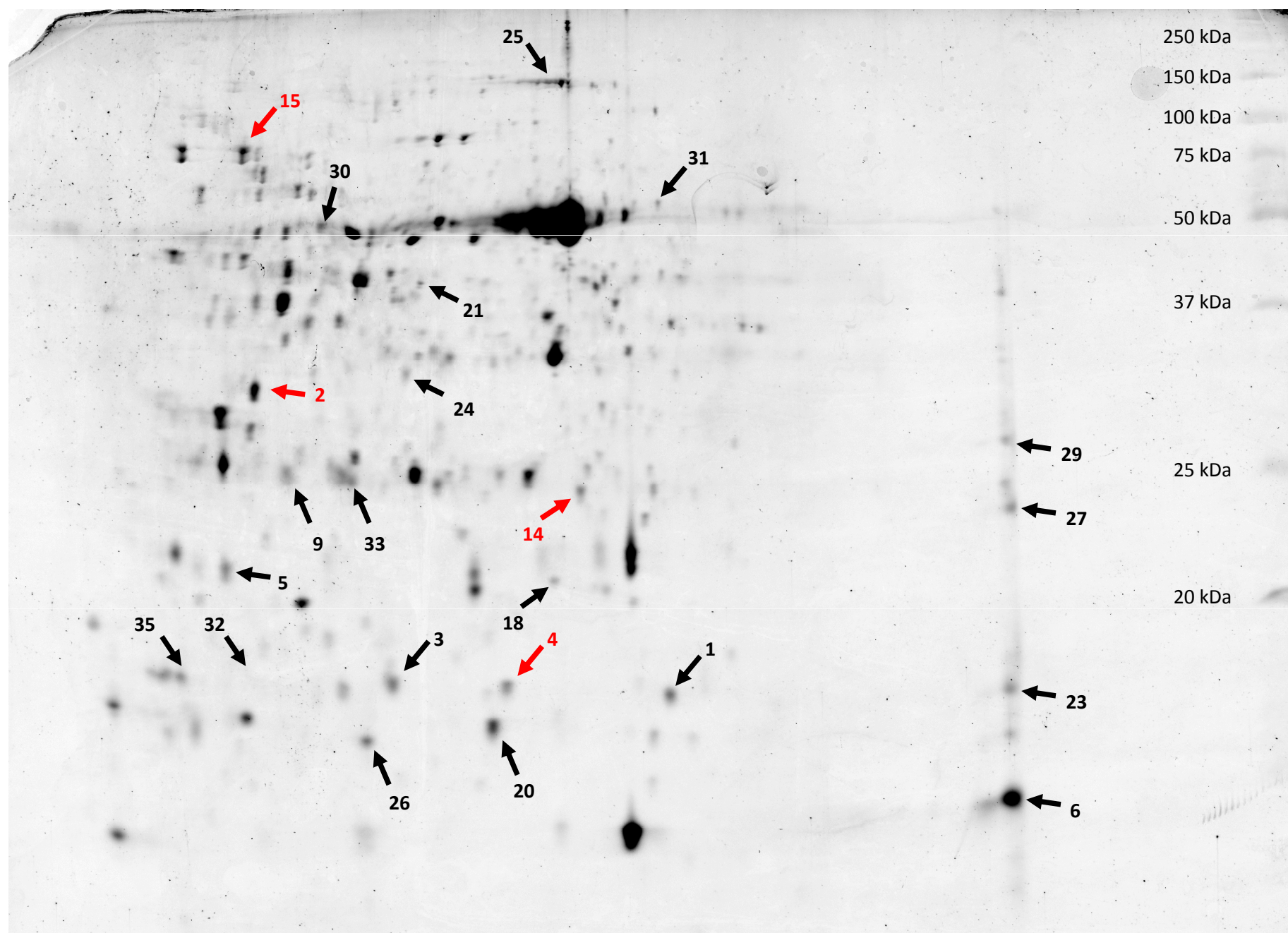

Supplement: Supplementary file 1 — Supplementary material 1 (PDF 1453 kb) [file 425_2014_2221_MOESM1_ESM.pdf]
