# Supplementary material for: Redox proteomics and physiological responses in Cistus albidus shrubs subjected to long-term summer drought followed by recovery
Source: Planta. 2014 Dec 13;241(4):803–22. doi: 10.1007/s00425-014-2221-0 (PMC4361772; doi:10.1007/s00425-014-2221-0)

Fig. S2 Spot images showing the variations of some identified proteins through the experiment
T_0, time 0; WW, well-watered; WS, water stressed; Water-stress recovery

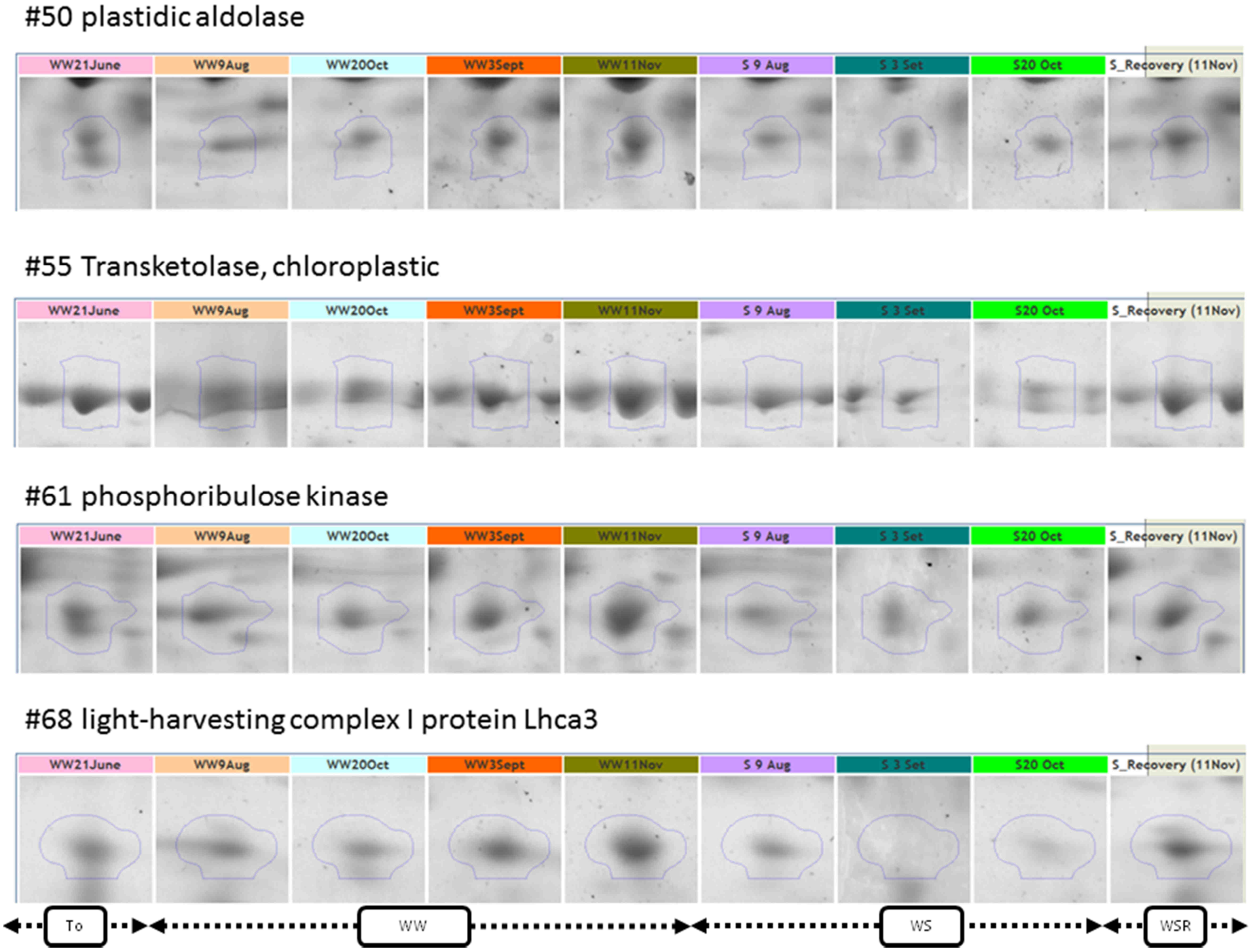

Supplement: Supplementary file 2 — Supplementary material 2 (PDF 936 kb) [file 425_2014_2221_MOESM2_ESM.pdf]
